# Monitoring Bacterial Conjugation by Optical Microscopy

**DOI:** 10.3389/fmicb.2021.750200

**Published:** 2021-10-04

**Authors:** Gerardo Carranza, Tamara Menguiano, Fernando Valenzuela-Gómez, Yolanda García-Cazorla, Elena Cabezón, Ignacio Arechaga

**Affiliations:** Departamento de Biología Molecular, Instituto de Biomedicina y Biotecnología de Cantabria (IBBTEC), Universidad de Cantabria–CSIC, Santander, Spain

**Keywords:** antibiotic resistance, bacterial conjugation, fluorescence microscopy, conjugative ATPases, T4SS

## Abstract

Bacterial conjugation is the main mechanism for horizontal gene transfer, conferring plasticity to the genome repertoire. This process is also the major instrument for the dissemination of antibiotic resistance genes. Hence, gathering primary information of the mechanism underlying this genetic transaction is of a capital interest. By using fluorescent protein fusions to the ATPases that power conjugation, we have been able to track the localization of these proteins in the presence and absence of recipient cells. Moreover, we have found that more than one copy of the conjugative plasmid is transferred during mating. Altogether, these findings provide new insights into the mechanism of such an important gene transfer device.

## Introduction

Horizontal gene transfer is the main pathway for the widespread dissemination of antibiotic resistance genes (Mazel and Davies, 1999; de la Cruz and Davies, 2000; Rice, 2009; von Wintersdorff et al., 2016; Koraimann, 2018). The three main mechanisms involved in horizontal gene transfer are transformation, phage transduction and conjugation (Popa and Dagan, 2011; Daubin and Szollosi, 2016). Conjugation is particularly relevant, as it is responsible of the transmission of large plasmid DNA molecules (Waters, 1999; Smillie et al., 2010). The transfer of conjugative DNA requires a sophisticated machinery to carry out DNA mobilization and mating pair formation (de la Cruz et al., 2010; Cabezon et al., 2015; Zechner et al., 2017).

In Gram negative bacteria, conjugation is initiated by a specific protein that recognizes a DNA sequence in the plasmid (origin of transfer) and, upon a nucleophilic cleavage, remains covalently bound to the DNA (Byrd and Matson, 1997; Chandler et al., 2013). This multi-domain protein, named relaxase, is a large protein that is transported across the membranes of donor and recipient cells bound to the DNA (Draper et al., 2005; Garcillan-Barcia et al., 2007). This is a particular challenging process considering the size of the protein substrate, which ranges from 900 to 1,800 kDa, as in the case of TraI, the relaxase of the conjugative plasmid F (Frost et al., 1994), which is a member of the IncF family. In the IncW plasmid R388, the cleavage reaction occurs via a nucleophilic attack by relaxase TrwC on the 5′-side of the DNA phosphate. This transesterification reaction results in a covalent linkage between protein and DNA (Guasch et al., 2003; Gonzalez-Perez et al., 2007). After the cleavage reaction, donor DNA synthesis begins from the 3′end of the cleaved strand, so the single stranded DNA copy (ssDNA) that is transferred to the recipient cell is replaced by the new synthesized DNA strand.

In this way, conjugative DNA is transferred across the membrane channel covalently bound to the relaxase protein. The process is carried out with the help of another ATPase known as coupling protein (Cabezon et al., 1997). This hexameric ATPase, named TrwB in plasmid R388, couples the energy released from ATP hydrolysis to ssDNA pumping through the secretion channel (Tato et al., 2005; Cabezon and de la Cruz, 2006). The nucleoprotein complex formed by the conjugative DNA and the relaxase are meant to cross the membranes of donor and recipient cell through a secretion channel known as Type IV Secretion System (T4SS). This is a large macromolecular complex formed by 11 different subunits that spans the inner and outer membranes of donor cells (Alvarez-Martinez and Christie, 2009; Christie et al., 2014; Cabezon et al., 2015). At the base of the channel there are two hexameric ATPases that participate both in the biogenesis of T4SS and in the transport of the nucleoprotein complex (Cascales and Christie, 2003; Atmakuri et al., 2004; Arechaga et al., 2008; Peña et al., 2012). These ATPases are VirB4 and VirB11, named TrwK and TrwD, respectively, in the R388 plasmid system. VirB4 protein is the largest and most conserved constituent of T4SS (Fernandez-Lopez et al., 2006; Guglielmini et al., 2013). This protein is essential for the assembly of the T4S pilus, playing a fundamental role in powering the system. VirB11 also plays an essential role in the first steps of the DNA translocation pathway (Atmakuri et al., 2004). It has been suggested that VirB11 acts as a molecular switch between pilus biogenesis and substrate transport (Ripoll-Rozada et al., 2013), but it is worth noting that is not found in all T4SS. F plasmids, for instance, do not code for a VirB11 homolog (Frost et al., 1994; Lawley et al., 2003).

In the last few years, much progress has been done in the understanding of the genetic contribution and molecular architecture of the different components of the conjugative system. However, there are still important open questions on how this process is actually occurring. Here, by using optical microscope methods, we provide direct evidence on the localization of the most important proteins that drive the conjugation process in *Escherichia coli*. We have labeled with fluorescent constructs the relaxase, the coupling protein and the largest ATPase of the T4SS in R388 plasmid, and we have found that the pattern of localization of these proteins change upon contact with recipient cells. Furthermore, we have been able to visualize the result of the conjugation process and we have gathered evidence that indicates that more than one copy of the conjugative plasmid ends up in the recipient cell.

## Materials and Methods

### Cloning of Fluorescent Fusion Proteins

Combinations of different fluorescent markers fusioned to *trwB*, *trwC*, and *trwK* genes were created using plasmid pIM09 as a template (I. Matilla, doctoral thesis). pIM09 contains a fusion of trwB, GFP, and kanamicine resistance genes cloned in a pBR322 derivative vector (Supplementary Figure 6). The FRT Kanamycin resistance cassette was obtained from pKD4 plasmid (Datsenko and Wanner, 2000). For TrwC fluorescent constructs, *trwC* gene was obtained from R388 plasmid by PCR, adding *Hin*dIII and *Xho*I restriction sites at both ends (oligonucleotides 1 and 2 from Supplementary Table 2). For TrwK constructs, oligonucleotides 3 and 4 with the same restriction sites were used (Supplementary Table 2).

In order to create different fluorescent variants, *mCherry*, *mKate2*, and *mEOS* genes were obtained from pROD25 plasmid (Reyes-Lamothe et al., 2008) (oligonucleotides 5 and 6), pBAD33_*mKate2* plasmid (Addgene) (Shcherbo et al., 2007) (oligonucleotides 7 and 8), and from a pRSETa_*mEos4b* vector (Paez-Segala et al., 2015) (Addgene) (oligonucleotides 9 and 10). In all cases, the fluorescent variants were inserted into *Xho*I and *BamH1* restriction sites from pIM09 plasmid. TrwBmCherry was also cloned in a pHis vector to estimate membrane co-location with the fluorescent dye Nonyl-Acridine Orange (NAO), which is a membrane specific stain.

Fluorescent fusion constructs in plasmid pIM09 were then amplified by PCR, by using oligonucleotides containing 50 bases that perfectly matched the flanking R388 sequence where the constructs had to be inserted. Oligonucleotides 11 and 12 were used for *trwB* constructs, oligonucleotides 13 and 14 for *trwC* constructs and oligonucleotides 15 and 16 for *trwK* constructs (Supplementary Table 2). The amplified fragments were purified in an agarose gel and transformed into a *E. coli* TB10 strain containing R388 plasmid for homologous recombination. Recombination was activated by growing cells at 42°C, as previously reported. Cells were plated on LB agar containing kanamycin (50 μg/ml) and trimethoprim (20 μg/ml) to select the recombinant R388 plasmids. The selected plasmids were subsequently analysed by sequencing.

### Bacterial Conjugation Assays

Conjugation donor strains were derivatives of *E. coli* K12 strain MG1655 carrying either a kanamycin-resistance derivative of plasmid R388 (plasmid pSU2007) or a R388 plasmid expressing the conjugative proteins TrwB, TrwC, and TrwK fused with different fluorescent tags under its natural promotor in plasmid R388. Overnight cultures of MG1655 cells carrying each of these constructs grown in LB medium were mated (1:1) with recipient strain UB1637 as described previously (Peña et al., 2011), with the exception of SeqA-GFP experiments, in which a MG1655 Dam methylase deficient strain (*Dam*^–^) (Palmer and Marinus, 1994) was used as a recipient. Cells were collected by centrifugation and resuspended in fresh LB medium. Samples were placed on Millipore filters (0.2 μm) for 1 h at 37°C. Then, the filter was washed in LB and resuspended. Dilutions (1:10 to 1:10^3^) were plated, selecting for transconjugant and donor cells with appropriate antibiotics. Transconjugants were selected on L-agar plates containing streptomycin (300 μg/ml) and kanamycin (50 μg/ml). Conjugation frequencies were calculated as a ratio between the number transconjugants versus donor cells.

### Standard Fluorescence Microscopy

For standard microscopy, microscope slides covered with melted agarose and framed with a Frame-Seal Incubation Chamber (Biorad) were used. M9 minimal media (200 μl) was mixed with low melting point agarose (1.5%, w/v) and added into the cavity formed by the adhesive frame. This chamber was protected with another microscope slide. Pads were cooled down for 30 min at room temperature. Seeding cultures of MG1655 cells, previously grown in M9 minimal media with glucose as carbon source (2 μl, 0.1 OD600_*nm*_), were spread onto circular agarose pads (6 mm) and, after drying, were covered with a coverslip. For conjugation experiments, MG1655 donor cells and UB1637 recipient cells were grown overnight in M9 minimal media. Next day, a 1:1,000 dilution was prepared and cells were grown until the optical density (OD600_*nm*_) reached a value of 0.6. Then, donor and recipient cells were mixed at a 1: 2 ratio in a final volume of 400 μl. Cells were then centrifuged and resuspended in 40 μl of M9 medium. Aliquots (2 μl) were spread onto a slice of agarose dissolved in minimal medium as indicated above, and the sample was incubated during 15 min at 37°C for mating.

Standard fluorescence microscopy was carried out using a Zeiss Axio Imager M1 upright fluorescence microscope (Zeiss Plan-Neofluar _100/1.30 NAOil Ph3 objective), equipped with a 12 bits B&W camera (AxioCam MRm), using a standard rhodamine filter unit (Ex. 546/12 – Em. 608/65). Green fluorescence of mGFP and mCitrine was detected by using a standard GFP filter unit (Ex. 470/40 – Em. 525/50). Membranes were visualized with Acridine Orange 10-nonyl bromide (NAO, Sigma-Aldrich). NAO fluorescence was also detected using the same GFP filter unit.

### Time Lapse and TIRF Microscopy

Bacterial cells were grown as indicated previously. Samples were placed onto a cover glass–bottom imaging dish, with the bacteria sandwiched between the agarose pads and the cover glass. The dish was placed into a custom stage insert, which holds the dish tightly. After sealing with Parafilm^*R*^ or grease, bacteria cells were seeded onto individual agarose pads and incubated for 10 min at 37°C.

Live conjugation was monitored in a Nikon Eclipse Ti2 microscope equipped with a Hamamatsu ORCA-Flash 4.0 camera using a CFI Apochromat TIRF100xc Oil objective. For TIRF imaging, a Nikon A1 confocal laser microscope equipped with Hamamatsu 9100-C2 camera and a Plan Apochromat TIRF 100x Oil DIC HN2 objective was used. Super-resolution imaging (d-STORM) was performed in an Olympus IX-73 microscope equipped with 100xoTIRF Olympus UAPON objective and an iXon ULTRA897 EMCCD camera (Andor). Image analysis was carried out using Fiji/ImageJ software (National Institutes of Health, United States).

### Membrane Fractionation and Immunodetection of the Fluorescent Conjugative ATPases

Cells were harvested by centrifugation at 4,000 × *g* and re-suspended in a buffer containing 50 mM Tris, pH 7.5, 0.5 mM EDTA, 0.1% PMSF. Cells were lysed in a TS cell disruptor (Constant Systems, United Kingdom) at 25 kpsi. Lysates were centrifuged at 800 × *g* (15 min), followed by another centrifugation at 10,000 × *g* (15 min) to remove unbroken debris. Membranes were collected by ultracentrifugation at 100,000 × *g* (30 min). The pellet (membrane fraction) was re-suspended in 50 mM Tris, pH 7.5, 0.5 mM EDTA, 0.1% PMSF, and 2% SDS. Protein samples were run in a SDS-PAGE, transferred to a nitrocellulose filter and incubated with rabbit antiserum (anti-TrwC, anti-TrwB, and anti-TrwK, respectively). Images were obtained after incubation with an IRDye anti-rabbit IgG (goat) antibody conjugate, using an Odyssey scanner (Li-Cor Biosciences).

## Results

### Localization of the Main Conjugative ATPases in Donor Cells in the Absence of the Host

Conjugative ATPases are key players in plasmid transfer. They assist in the processing and transport of conjugative DNA from donor to recipient cells. In order to assess the subcellular location of these proteins, different fluorescent tags were attached to the main three ATPases involved in the conjugative process of plasmid R388, our model system.

The first step in conjugation is the formation of the relaxase/DNA complex that will be transferred to the recipient cell. The relaxase is a large protein with two functional domains: a N-terminal domain, that produces the nick on the DNA, and a C-terminal helicase domain involved in the processing of the DNA (Grandoso et al., 1994). In R388 plasmid, the relaxase protein is TrwC. Based on biochemical and structural information, we have generated a repertoire of fusion proteins at the C-terminus of TrwC with several fluorescent tags (mGFP, mCherry, mEos4, and mKate2), cloned directly in the conjugative R388 plasmid, under its natural promotor. We followed a similar strategy with the two other main ATPases of the system: TrwB, also known as the coupling protein, which is involved in the transport of the DNA (Cabezon and de la Cruz, 2006), and TrwK, a large ATPase (Arechaga et al., 2008) localized at the base of the T4SS channel (Atmakuri et al., 2004; Hu et al., 2019). These ATPases are the VirD4- and VirB4-homologs in the *Agrobacterium tumefaciens* system, respectively (Atmakuri et al., 2004; Cabezon et al., 2015). In both cases, the fluorescent tag was also inserted at the C-terminus. TrwB (VirD4) is a membrane protein with two transmembrane α-helices at the N-terminal end, and TrwK (VirB4) is bound to the transmembrane protein TrwM (VirB3) also by the N-terminus (in some VirB4 homologs both proteins are fused as only one protein) (Arechaga et al., 2008). Therefore, the best location for the fluorescent tags was, in all cases, the C-terminal ends.

**FIGURE 1:**
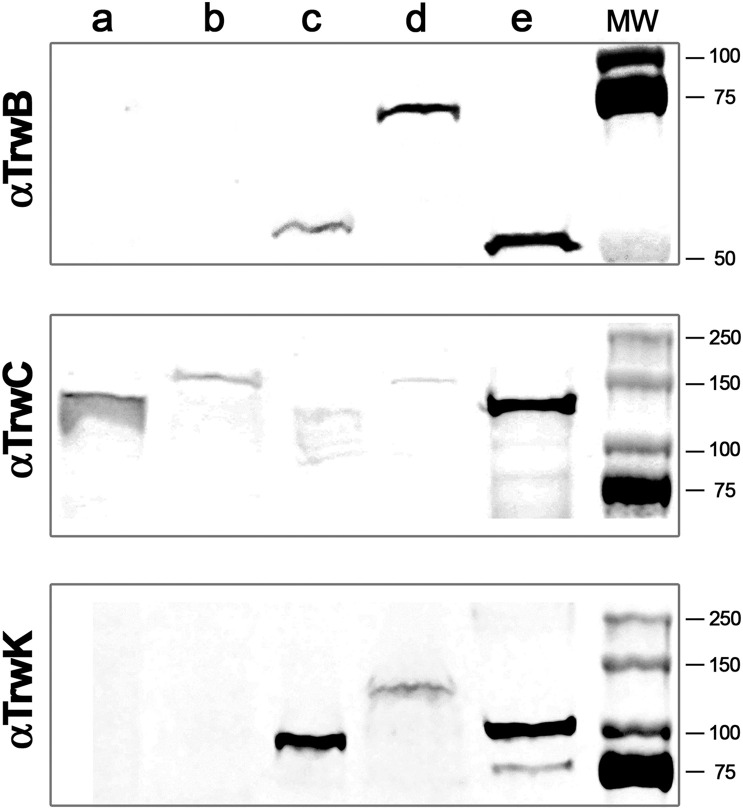
Immunodetection and subcellular localization of conjugative ATPases. MG1655 *Escherichia coli* cells were transformed with R388 wild type plasmid or with R388 encoding for TrwBmKate2, TrwCmKate2, or TrwKmKate2. Cells were grown to stationary phase and harvested by centrifugation. Cell pellets were re-suspended and lysed by mechanical methods. Soluble and membrane fractions were separated by ultra-centrifugation. Aliquots of each fraction were run in a SDS-PAGE. Gels were transferred to a nitrocellulose membrane and incubated with antibodies anti-TrwB (*upper panel*), anti-TrwC (*middle panel*), or anti-TrwK (*bottom panel*) and then revealed with IRDye. *Upper panel*: (a) wild type R388 soluble fraction, (b) R388_TrwBmKate2 soluble fraction, (c) wild type R388 membrane fraction, (d) R388_TrwBmKate2 membrane fraction, and (e) purified TrwBΔN70 protein. *Middle panel*: (a) wild type R388 soluble fraction, (b) R388_TrwCmKate2 soluble fraction, (c) wild type R388 membrane fraction, (d) R388_TrwCmKate2 membrane fraction, and (e) purified TrwC protein. *Bottom panel*: (a) wild type R388 soluble fraction, (b) R388_TrwKmKate2 soluble fraction, (c) wild type R388 membrane fraction, (d) R388_TrwKmKate2 membrane fraction, and (e) purified TrwK protein. Estimated MW values for TrwB, TrwBmKate2, and TrwBΔN70 proteins are 56.3, 82.4, and 48.6 kDa, respectively. Estimated MW values for TrwC and TrwCmKate2 proteins are 107.4 and 133.5 kDa, respectively. Estimated MW values for TrwK and TrwKmKate2 proteins are 93.8 and 119.9 kDa, respectively.

We checked that the fluorescent tags did not hamper the *in vivo* function of these proteins in mating assays, which allowed us to conclude that fusion proteins of TrwC, TrwB, and TrwK with various fluorescent labels (GFP, mCherry, mKate2, or mEos4) were functional for R388 plasmid transfer, although with lower conjugation frequencies than the wild type plasmid (Supplementary Table 1). Moreover, expression of the three fluorescent ATPases was also checked by immunodetection assays (Figure 1). The subcellular location of the three fluorescent variants in donor cells was similar to that expected in the wild type version. Thus, we were able to determine that TrwCmKate2, TrwBmKate2, and TrwKmKate3 were expressed and targeted in a way similar to wild type TrwC, TrwB, and TrwK, respectively. TrwB and TrwK were associated to the membrane fraction and TrwC was found mainly in the cytoplasm, as expected.

**FIGURE 2:**
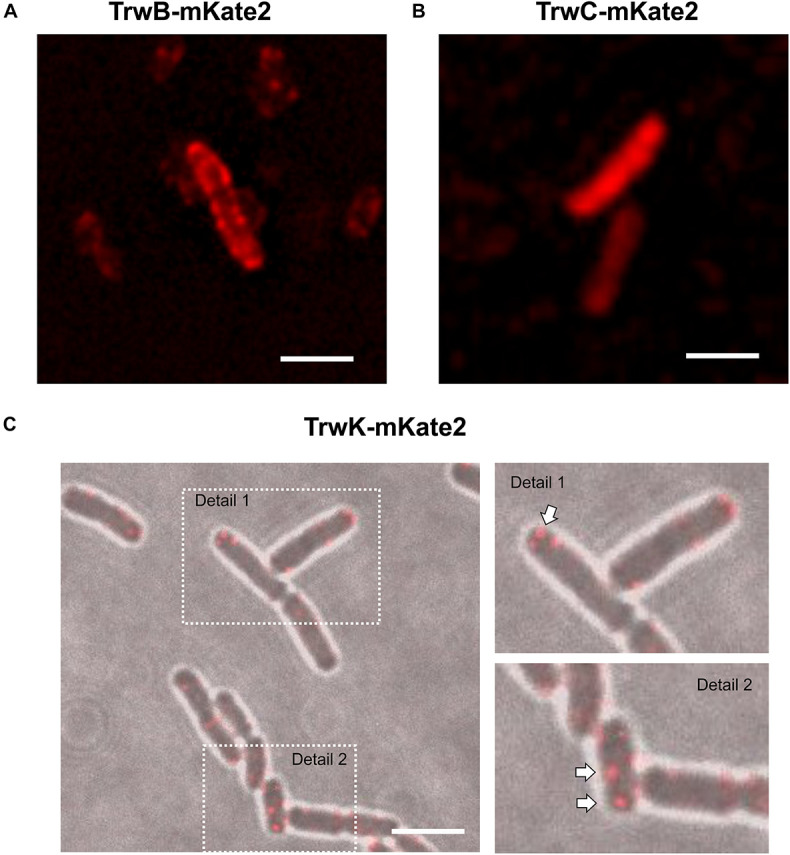
Localization of conjugative proteins in donor cells in the absence of recipients. MG1655 cells expressing TrwBmKate2 **(A)**, TrwCmKate2 **(B)**, and TrwKmKate2 **(C)** were grown in M9 minimal media and examined by fluorescent microscopy. In all cases, fusion proteins are expressed under its respective natural promoter in plasmid R388. TrwBmKate2 was widely distributed in the membrane **(A)** (see also Supplementary Movie 1), TrwCmKate2 was dispersed in the cytoplasm **(B)** and TrwKmKate2 was found forming discrete foci (around six per cell) **(C)**, which might indicate the number of secretion systems assembled. In this last case, phase contrast/color bright field images are shown to better visualize both the foci, and the bacterial cells. (Scale bar: 2 μM).

Next step was to determine by optical microscopy the localization of these proteins before and during conjugation. TrwB, which is a membrane protein, when fused to mKate2, was forming fluorescent foci randomly localized at the periphery of the donor cells (Figure 2A, Supplementary Movie 1, and Supplementary Figure 1). This membrane localization of TrwB was also confirmed with a TrwBmCherry fluorescent variant by using the fluorescent dye Nonyl-Acridine Orange (NAO), which is a membrane specific stain (Supplementary Figure 2). A similar membrane localization pattern was observed by super-resolution d-STORM microscopy in cells in which TrwB was fused to mGFP (Supplementary Figure 3). In contrast, TrwC, which is a soluble protein, was widely distributed along the cytoplasm (Figure 2B), regardless of the different fluorescent constructs.

In the absence of recipient cells, the high number of TrwB foci observed indicated that the system is unconstrained (Supplementary Figure 1 and Supplementary Movie 1). Likewise, the number of TrwC foci in the absence of recipient cells and their localization, widely disseminated across the cell, enforced this idea. In contrast to TrwB and TrwC, the number of TrwK foci was limited to ∼6 foci/cell (Figure 2C). TrwK is an essential part of the T4SS, which ensembles as a hexamer or as double hexamer at the base of the channel (Arechaga et al., 2008; Low et al., 2014). Therefore, it might be a good indicative to evaluate the number of Type IV transport systems per cell. The membrane localization of TrwKmKate2 was also confirmed by TIRF microscopy (Supplementary Figure 4), since foci localized in the membrane can be visualized by this technique. These results indicate that TrwB and TrwC proteins remain widely distributed across the bacterial cell in the absence of receptors, whereas the assembly of T4SS is limited to a few copies (around six per bacterial cell).

### Pattern Distribution Upon Contact With the Recipient Cell

**FIGURE 3:**
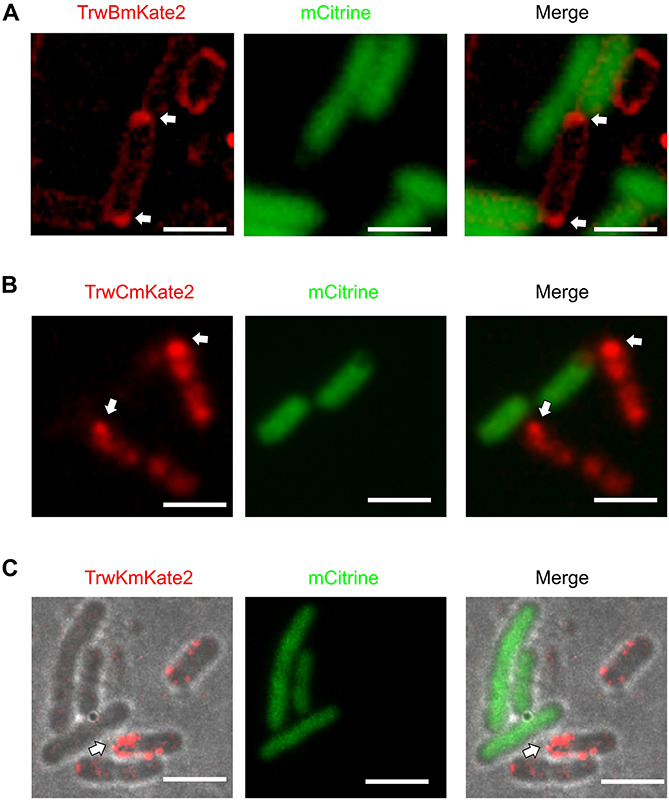
Localization of conjugative proteins in the presence of recipient cells. MG1655 donor cells expressing TrwBmKate2 **(A)**, TrwCmKate2 **(B)**, and TrwKmKate2 **(C)** under their respective natural promotors in R388 plasmid were mixed with UB1637 recipient cells expressing the fluorescent protein mCitrine. Fluorescence of mKate2 protein derivatives in donors (red) and mCitrine (green) in recipients is shown on the left and central panels, recorded at 630 and 540 nm, respectively. The right panel shows the merged images. Upon contact between both donor and recipient cells, the conjugative fluorescent proteins localize at the poles, on those specific areas that are in close contact with a recipient cell. In panel C, phase contrast/color bright field images are shown to better visualize both, the foci and the bacterial cells. The arrow shows the areas in the donor cell where the conjugative ATPases are compiled upon contact with the recipient cell. [Scale bars: 2 μM **(A)** and 3 μM **(B,C)**].

Next, we studied the localization of these ATPases upon contact with the recipient cells to determine if the distribution of these proteins changed during the conjugative process. In these experiments, TrwB, TrwC, and TrwK proteins fused to mKate2 and placed in R388 plasmid under the natural promoters (*PtrwA* for *trwB* and *trwC* and *PkorA* for *trwK*) were expressed in donor cells, whereas green fluorescent proteins (mGFP or mCitrine) were expressed in the recipient cell. After incubating both donor and recipient cells during 1 h, images were acquired. Inspection of the distribution pattern of TrwBmKate2 and TrwCmKate2 showed a dramatic change in the localization of these proteins (Figure 3), as compared with that observed in the absence of recipient cells (Figure 2) (see also fluorescent intensity profiles in Supplementary Figure 5). In the presence of recipient cells, TrwBmKate2 localized close to the poles, in contrast to the random distribution on the membranes in its absence. Even more dramatic was the change in the localization pattern of TrwCmKate2. In the presence of recipient cells, TrwCmKate2 formed defined foci, mainly localized at the poles (Figure 3B), whereas in its absence, TrwCmKate2 was widely distributed in the cytoplasm (Figure 2B). These changes in the localization pattern were also identified in the case of TrwKmKate2 (Figure 3C), with a tendency to migrate to the pole cells upon contact with recipients.

### Monitoring Bacterial Conjugation

**FIGURE 4:**
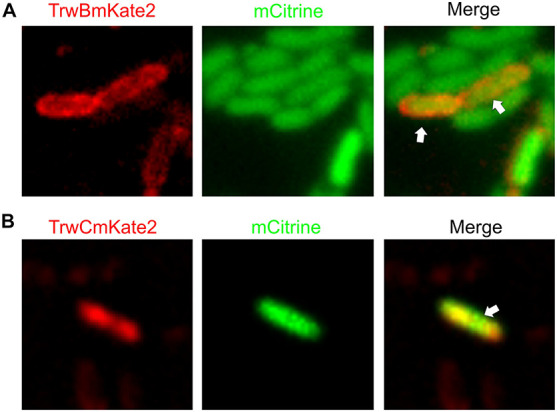
Visualization of transconjugant cells. MG1655 donor cells hosting R388:*trwBmKate2* **(A)** or R388:*trwCmKate2* **(B)** were mixed with UB1637 recipient cells expressing mCitrine. Conjugation was performed in enriched M9 minimal media plates and then, cells were transferred to the microscope for image-recording. Images were recorded at different emission wavelengths (540 and 630 nm, for mCitrine and mKate2, respectively) at the same location, in order to discriminate donor, recipient and transconjugant cells. Merged images show the transconjugant cells in orange (*right panel*). These transconjugants are UB1637 cells expressing mCitrine that, after receiving the R388 plasmid, have started to express TrwBmKate2 **(A)** or TrwCmKate2 **(B)**, encoded by the plasmid.

Once the localization of the different proteins was determined both in the presence and absence of recipient cells, the next step was trying to obtain live images of bacterial conjugation. Donor and recipient cells with different fluorescence markers were incubated together and plated on the microscope slides. However, although many different strategies were followed, we were unable to monitor *in situ* nucleoprotein transfer by placing donor and recipient cells directly under the microscope. As previously mentioned, fluorescent variants were found to be functional in mating assays. The insertion of the fluorescent tags into the R388 plasmid only decreased transfer efficiency by 1–2 logs in plates (Supplementary Table 1). In fact, when cells expressing TrwBmkate2 or TrwCmkate2 were mated and transferred immediately to the pad, transconjugants were observed, regardless of the fluorescent tag (Figure 4). As discussed later, conjugation on the pad seems to be inhibited by unknown reasons, despite temperature and atmospheric CO_2_ conditions were kept constant.

**FIGURE 5:**
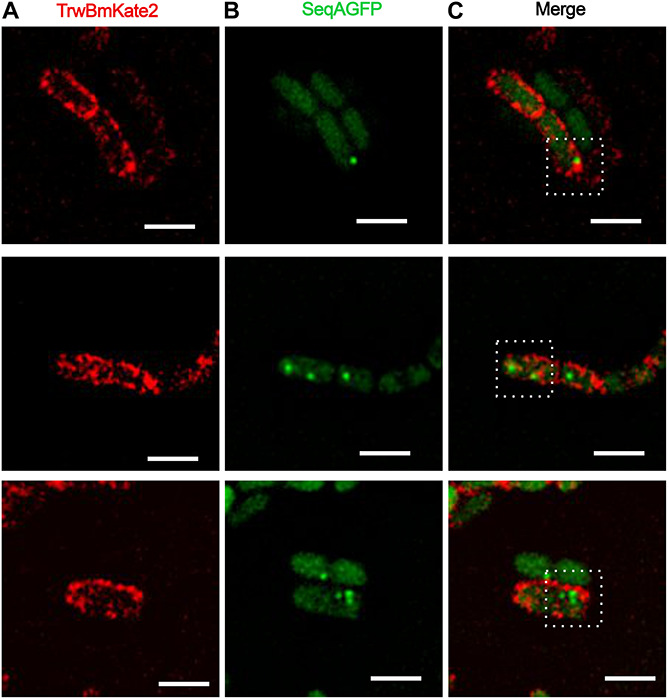
Conjugative R388 transfer visualized as fluorescent SeqA-GFP foci in recipient cells. MG1655 *dam*^+^ donor cells hosting R388:*trwBmKate2* were mated with *dam*^–^ recipient cells expressing SeqA-GFP from the chromosome. Conjugation was performed in enriched M9 minimal media plates and then, cells were transferred to the microscope for image-recording. Transconjugants were monitored by the presence of fluorescent foci, indicating the binding of SeqA-GFP to hemy-methylated DNA. In some transconjugant cells, up to three independent foci were detected. Images show the same pad of transconjugant cells recorded at different emission wavelengths: 630 nm for mKate2 (**A**, red) and 540 nm for SeqA-GFP (**B**, green). The merged images in panel **(C)** show the fluorescent foci of SeqA-GFP plus TrwBmKate2 in the membrane, which is expressed from the recently transferred R388 plasmid. (Scale bar: 2 μM).

In order to circumvent this problem, we decided to study R388 conjugation by monitoring the fluorescence of the protein SeqA fused to GFP in the recipient cell (SeqA-GFP), following a strategy similar to that used with plasmid F (Babic et al., 2008). SeqA is a protein that binds to hemi-methylated DNA (Lu et al., 1994; Brendler and Austin, 1999), preferentially to newly replicated DNA, with high affinity in cells producing Dam methylase. In Dam methylase deficient cells (*Dam*^–^) (Palmer and Marinus, 1994) this binding is prevented, and fluorescence of SeqA is undetectable. Following this strategy, we mated *Dam*^+^ donor cells harboring R388:*trwBmKate2* with *Dam*^–^ recipient cells in which the seqA-GFP fusion construct was inserted in the chromosome under the control of the native seqA promoter, replacing the seqA gene. Once a R388 plasmid single DNA strand is transferred from a donor cell (with methylase activity) to a recipient cell, a copy of complementary non-methylated DNA will be synthesized to generate a hybrid hemi-methylated DNA, promoting SeqA-GFP binding. Therefore, conjugation events would be directly related to the number of green fluorescent foci observed in transconjugant cells. As expected, when *Dam*^–^ donor cells were used for conjugation, no fluorescent foci were observed in the recipient cells (data not shown). Upon complementation, transconjugants start to express TrwBmKate2 protein, which could be identified as cells expressing TrwBmKate2 plus the SeqA-GFP foci (Figure 5). Surprisingly, in some transconjugant cells (∼16%), we were able to observe more than one green foci (Figures 5B,C), which implies that more than one copy of the R388 plasmid has been transferred. The implications of this finding will be discussed lately.

## Discussion

Since the discovery of bacterial conjugation as the main mechanism of horizontal gene transfer (Lederberg and Tatum, 1946), great advances have been done in the understanding of the genetic mechanisms driving this process (Frost and Koraimann, 2010; Cabezon et al., 2015; Hu et al., 2019). Direct observation of horizontal gene transfer has been previously reported for F plasmid (Babic et al., 2008), which uses a long pilus to mate in liquid media (Paranchych and Frost, 1988; Clarke et al., 2008). However, many questions about how this process is really happening remain open. For instance, it is not clear yet how DNA is transferred, which is the signal that triggers the process, or even how the bacterial cells respond to that elusive signal. Here, trying to shed light to some of these questions, we have cloned the relaxase, the coupling protein, and the biggest and most conserved T4SS component (TrwK/VirB4) fused with a repertoire of fluorescent probes to visualize the different stages associated to the conjugative process. Although several experiments with fluorescent conjugative proteins have been previously reported (Clarke et al., 2008; Bauer et al., 2011; Arends et al., 2012; Segura et al., 2014; Nolivos et al., 2019), there is not a description of the localization of the proteins involved in each stage of the conjugative process. Furthermore, previous reports were mainly carried out with the fluorescent fusion proteins cloned in artificial expression vectors. It is known that the over-expression of fluorescent proteins under those circumstances may produce experimental artifacts, including protein aggregation or saturation of protein targeting machinery, leading to inappropriate localization (Day and Davidson, 2009). In order to prevent these problems, in this work, functional fluorescent proteins were expressed from its native promoter in plasmid R388.

After checking that the fluorescent fusions were functional, the next step was to localize these proteins in bacterial donor cells in the absence of recipients. We observed that, under these conditions, the number of copies of the coupling protein and the relaxase was much higher than the number of copies (around 6) of the conjugative R388 plasmid (Guynet et al., 2011). This result indicates that, even in the absence of receptor cells, *dtr* (DNA processing and transfer) genes are being expressed. However, *trwk* gene, which is part of the *mpf* (mating pore formation) operon, is more strictly downregulated, as judged by the low number of foci observed (around 6). As previously shown (Fernandez-Lopez et al., 2014), when the full regulatory network of the R388 is present, all plasmid promoters are repressed. However, it is important to note that *dtr* and *mpf* genes have distinctive transcriptional regulators (Fernandez-Lopez et al., 2014) and, therefore, a differential transcription of these genes could result in different expression levels, as observed in this work.

There are also differences in the localization pattern of these proteins (Supplementary Figure 5). In the absence of recipient cells, the coupling protein TrwB is uniformly dispersed along the membrane, whereas the relaxase is widely spread in the cytoplasm (Figure 2). TrwK, the main ATPase of the T4SS is always located bound to the membrane (Figure 2C), thanks to its interaction with TrwM, an integral membrane protein (Arechaga et al., 2008). TrwK is essential for the assembly of the T4S pilus. It docks onto the base of the secretion channel (Low et al., 2014) and plays a fundamental role in powering the assembly and function of the T4SS. Therefore, TrwK foci would be a good indicator of the number of secretion systems assembled in the donor cell. This is in agreement with data published previously (Gilmour et al., 2001), in which an average of five to six fluorescent foci were observed in the membrane of cells expressing TrhC protein (the VirB4-homolog from R27 plasmid), fused to GFP at the C-terminus (Gilmour et al., 2001; Gunton et al., 2005). Interestingly, these authors showed that most of the R27 transfer proteins were required for focus formation (Gilmour and Taylor, 2004).

In this work we have also observed that the localization of conjugative ATPases changed dramatically upon contact with the recipient cells. Under these conditions, these proteins tend to migrate to the poles of the cell (Figure 3), specifically to those areas in close contact with a recipient cell. This result indicates that such a contact triggers some sort of signal that dictates the reorganization of the conjugative ATPases. This is particularly evident in the case of the coupling protein TrwB and in TrwC.

In a next step, we focused on trying to observe bacterial conjugation directly on the pad, under the microscope light. However, under our experimental conditions, we were unable to observe cells mating on the microscope pad. Bacterial cells that in normal circumstances are able to conjugate, seem to stop mating as soon as they are placed on the microscope pad. Several hypotheses could explain this observation, based on a physical perturbation of the bacterial cells. The light of the microscope might be inhibiting the conjugation process. Alternatively, it might be possible that the pad of agar in which the cells are embedded is not ideal for bacterial conjugation. To circumvent this problem, we opted for mating donor and recipient cells in enriched solid M9 minimal media and, then, transfer the cells to the microscope pad for image recording. By using this approach, we were able to obtain images of transconjugants (Figure 4). These transconjugants can be identified as receptors expressing m-Citrine that have also received an R388 plasmid that expresses either TrwBmKate2 (4A) or TrwCmKate2 (4B) fusion proteins.

Additionally, we opted for monitoring the onset of transconjugants by using as receptors *dam*^–^ cells harboring the *seqA* gene fused to GFP. SeqA is a protein that only binds to hemi-methylated DNA (Brendler and Austin, 1999; Waldminghaus and Skarstad, 2009). When a methylated copy of the R388 plasmid enters the recipient cell, a complementary non-methylated strand will be generated by the host. Under these conditions, SeqA-GFP is able to bind to the DNA and fluorescence can be recorded. Similar experiments were previously described to monitor horizontal gene transfer in *E. coli* mediated by the F pili (Babic et al., 2008) or in the gram positive *Bacillus subtilis* (Babic et al., 2011). In the case of F pili, conjugation events were visualized between cells separated as far as 12 μM, although in some cases cell-to-cell contacts were also observed. This is due to the dynamics of the thin, flexible F pili (Clarke et al., 2008), which can undergo cycles of retraction and extension forced by the addition or separation of subunits at the cell proximal end. Here, however, we deal with a very short, rigid pilus prepared for conjugation within solid media, which is characteristic of IncW plasmids. Therefore, cell-to-cell contact is the most likely scenario.

**FIGURE 6:**
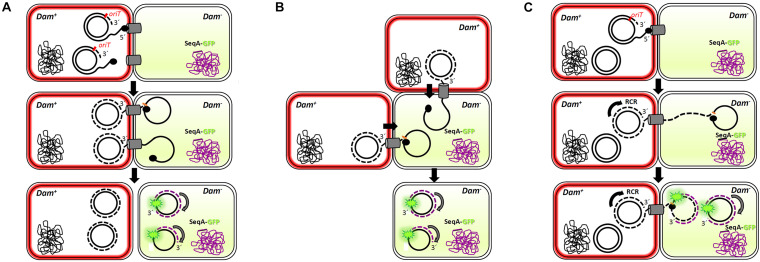
Multi-transfer of conjugative R388 plasmid. The passage of more than one copy of the conjugative plasmid from donor to recipient cells, as shown by the presence of more than one foci in the recipient cell (Figure 5), is compatible with three models. Dam^+^ donor cells contain R388 plasmid copies that express TrwB-mKate2 protein, coloring the membrane in red. Dam*^–^* recipient cells express SeqA-GFP protein from the chromosome, under the control of the native SeqA promoter. Methylated DNA is represented in black, whereas no methylated DNA is in purple. SeqA-GFP expression in the recipient cell is diffuse in the absence of hemi-methylated DNA. Only when a methylated copy of the plasmid is transferred and DNA replication starts in the recipient cell, SeqA-GFP is able to form compact foci. In model 1 **(A)**, conjugation might occur simultaneously from two or more secretion systems in contact with the same recipient cell, giving rise to at least two fluorescent foci. In model 2 **(B)**, two donor cells would transfer the plasmid simultaneously to the same recipient cell. In model 3 **(C)**, only one secretion system is functional but, since the transfer of plasmid ssDNA is accomplished of rolling circle replication (RCR) in the donor cell, multiple single-stranded linear copies of the plasmid (a concatemer) would be transferred bound to the relaxase TrwC. Once in the recipient cell, a new cleavage by TrwC on the *nic* site would re-circularize the plasmid DNA, resolving as many plasmid copies as those present in the concatemer. The synthesis of the complementary non-methylated strand would be associated to SeqA-GFP binding, observed as fluorescent foci.

Interestingly, and similarly to what was observed for the F-plasmid in previous reports (Babic et al., 2008), more than one foci could be imaged in some transconjugant cells (Figure 5). This result implies that more than one copy of the conjugative plasmid has been transferred to the recipient cell, which, *a priori*, is in contradiction with the incompatibility exclusion principle. A plausible explanation might be that there is a lag between the entrance of the conjugative plasmid and the expression of the entry exclusion factor (Garcillan-Barcia and de la Cruz, 2008). On this basis, although a splitting of the hemi-methylated DNA segments during plasmid replication cannot be entirely ruled out, three different scenarios could be foreseen (Figure 6). In a first scenario, conjugation might occur simultaneously through two or even three secretion systems in contact with the same recipient cell. Consistent with this idea is the observation of several T4SS assembled in donor cells. Alternatively, two different donor cells might contact simultaneously the same receptor cell. In a third scenario, only one secretion system from a single donor cell would be involved. In this case, upon DNA cleavage by TrwC, DNA synthesis by rolling circle replication (RCR) would produce multiple single-stranded linear copies of the plasmid (a concatemer). While DNA synthesis still goes on in the donor cell, the relaxase would be transferred covalently bound to the resultant concatemer. Once in the recipient cell, a new cleavage by TrwC on the *nic* site would re-circularize the plasmid DNA, resolving as many plasmid copies as those present in the concatemer (Figure 6). Once the plasmid DNA copies have been processed in the recipient cell, the transferred lagging strands will be replicated as complementary non- methylated DNA, which would explain the existence of more than one focus in the host. This scenario is supported by previous work (Gonzalez-Perez et al., 2007), in which it has been proposed that tyrosine 18 in TrwC is responsible for the initiation of replication at *oriT* in the donor cell, while tyrosine 26 promotes termination of leading strand replication in the recipient cell.

## Conclusion

In this work, bacterial conjugation has been characterized by fluorescence microscopy. By labeling the main ATPases that drive conjugative DNA processing and substrate transport across Type IV secretion systems, we have been able to follow dynamic changes in its localization pattern upon contact with recipient cells. These results provide a better understanding of bacterial conjugation.

## Data Availability Statement

The original contributions presented in the study are included in the article/Supplementary Material, further inquiries can be directed to the corresponding authors.

## Author Contributions

GC, TM, FV-G, and YG-C performed the experiments. IA designed the work. EC and IA supervised the work and wrote the manuscript. All authors contributed to the article and approved the submitted version.

## Conflict of Interest

The authors declare that the research was conducted in the absence of any commercial or financial relationships that could be construed as a potential conflict of interest.

## Publisher’s Note

All claims expressed in this article are solely those of the authors and do not necessarily represent those of their affiliated organizations, or those of the publisher, the editors and the reviewers. Any product that may be evaluated in this article, or claim that may be made by its manufacturer, is not guaranteed or endorsed by the publisher.
