# Supplementary material for: Monitoring Bacterial Conjugation by Optical Microscopy
Source: Front Microbiol. 2021 Oct 4;12:750200. doi: 10.3389/fmicb.2021.750200 (PMC8521088; doi:10.3389/fmicb.2021.750200)
Supplement: Supplementary file 1 [file Data_Sheet_1.PDF]

TABLE S1. Transfer frequencies of R388 and R388 derivatives containing different fluorescent fusion proteins

	Conjugation Frequency	
Plasmid in donor [a]	Transconjugants/ml	Transconjugants/donor
R388	1.7×10^{8}	4.5×10^{-1}
R388::*trwBmCherry*	2.4×10^{7}	4.6×10^{-2}
R388::*trwBmEOS*	1.1×10^{7}	3.3×10^{-2}
R388::*trwBmKate*	3.7×10^{7}	6.4×10^{-2}
R388::*trwCmCherry*	4.5×10^{6}	5.6×10^{-3}
R388::*trwCmKate*	4.9×10^{6}	4.7×10^{-3}

[a] Donor cells (*E.coli* K12 strain MG1655) carrying the plasmids shown in the first column were mated with strain UB1637, as described in M&M. Conjugation frequency is shown as the number of transconjugants obtained per donor and transconjugants/ml. Data are the mean of at least four experiments.

TABLE S2. Oligonucleotides used for the cloning of fluorescent fusion proteins

Primers	Sequence (5’-3’)
1	CGCAAGCTTACTACGGGAGAGGACATGCTCAGTCACATGGTATTGACC
2	GCGCTCGAGCCTTCCGGCCTCCATGCCGCGCTCGGC
3	CGCAAGCTTACTACGGGAGAGGACATGGGGGCAATTGAATCCCGC
4	GCGCTCGAGTACGTCGCTCCTTTCGGCTTTC
5	AAACTCGAGGGTTCCGGAGTGAGCAAGGGCGAGGAGGATAAC
6	TTTGGATCCTTACTTGTACAGCTCGTCCATGCCACCGG
7	AAACTCGAGGGTTCCGGAGTGTCTGAGCTGATTAAGG
8	TTTGGATCCTTATCTGTGCCCCAGTTTGCTAGG
9	AAACTCGAGGGTTCCGGAGTGAGTGCGATTAAGCCAGAC
10	TTTGGATCCTTATCGTCTGGCATTGTC
11	GAGCGCGTGCGCGAGCGCGTCGTGATGCCGGCG
12	TGTCGGGTCAATACCATGTGACTGAGCATTAGATAGTCCCCTCAACGACATGGGAATTAGCCATGGTCC
13	CGGGGAGGCCGTAGCGCCGCAGCAGAGCGCCAGAGGGAGC
14	AAGAAAGCCCGTAGCACGCGCTACGGGCTTTTTCTTGTCCCTGCTTAGACATGGGAATTAGCCATGGTCC
15	GAAAGCATTATTGCCGAAGTCGG
16	AATGGCGGCCACCGCCGCCGTCATAACCAGCTTCTGACATGGGAATTAGCCATGGTCC

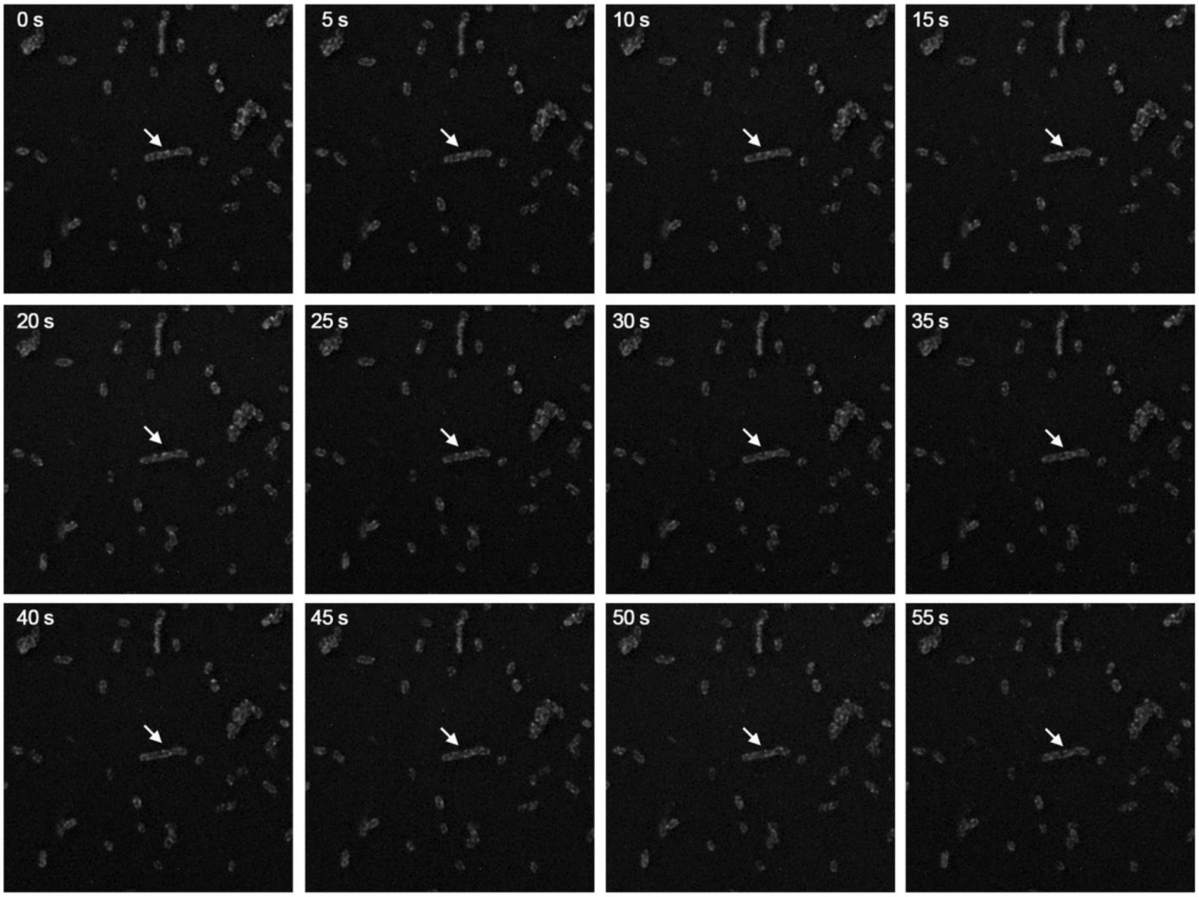

Figure S1. Localization of TrwB in the absence of receptor. Donor *E. coli* cells expressing TrwBmKate2 fusion protein from its native promotor in plasmid R388 were grown and placed on a microscopy slide embedded with M9 enriched minimal medium. Live images were recorded for several minutes. A sequence from still images taken at intervals of 5 seconds is shown.

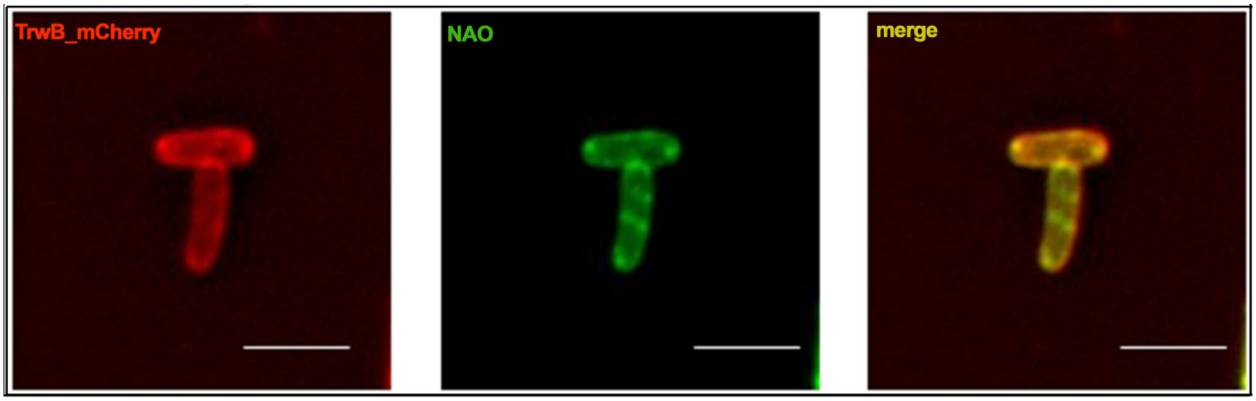

Figure S2. Membrane localization of TrwB. The coupling protein TrwB, fused with mCherry (red) and cloned in a pHis vector, co-localizes with the membrane probe NAO (green), which is an indication of the membrane localization of this protein.

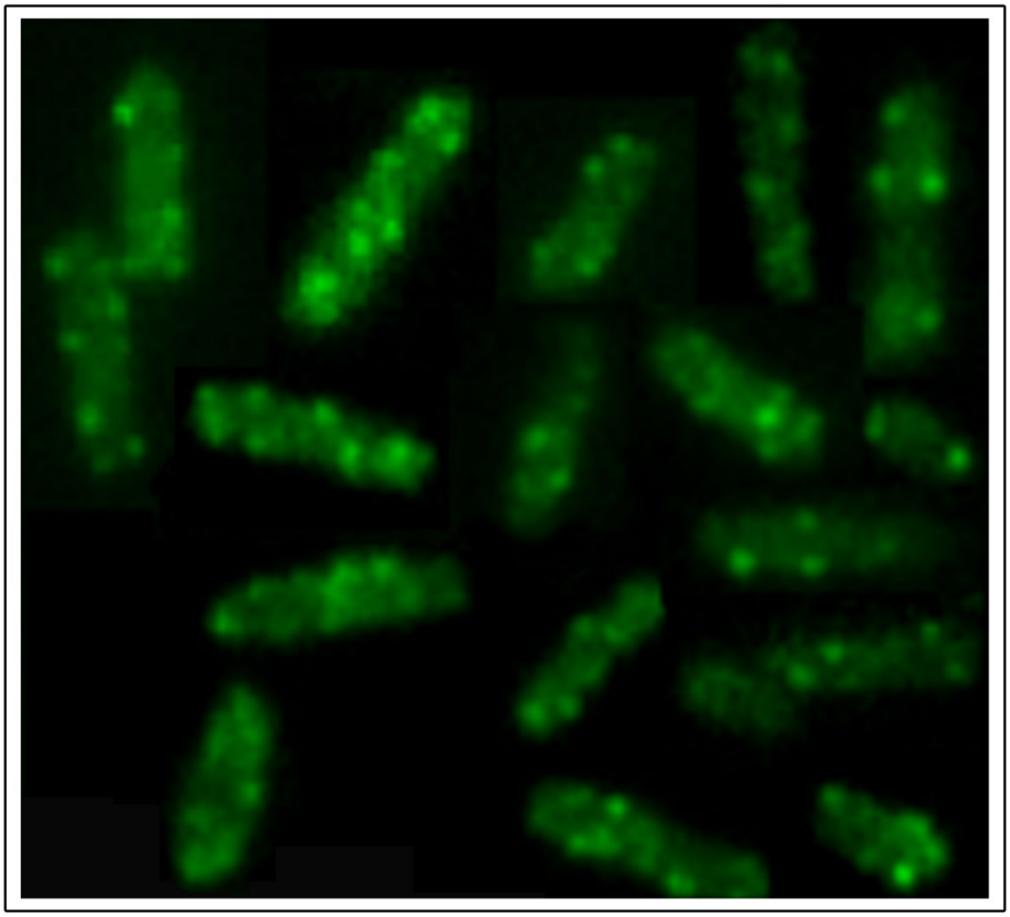

Figure S3. STORM images of R388_TrwBGFP. Gallery of representative images of TrwBGFP expressed from its native promoter in plasmid R388. Images were taken with an Olympus IX-73 microscope equipped an iXon ULTRA897 EMCCD camera (Andor) at 16 µm pixel size by using a 100xTIRF Olympus UAPON objective (NA= 1.49). Images were processed with ImageJ.

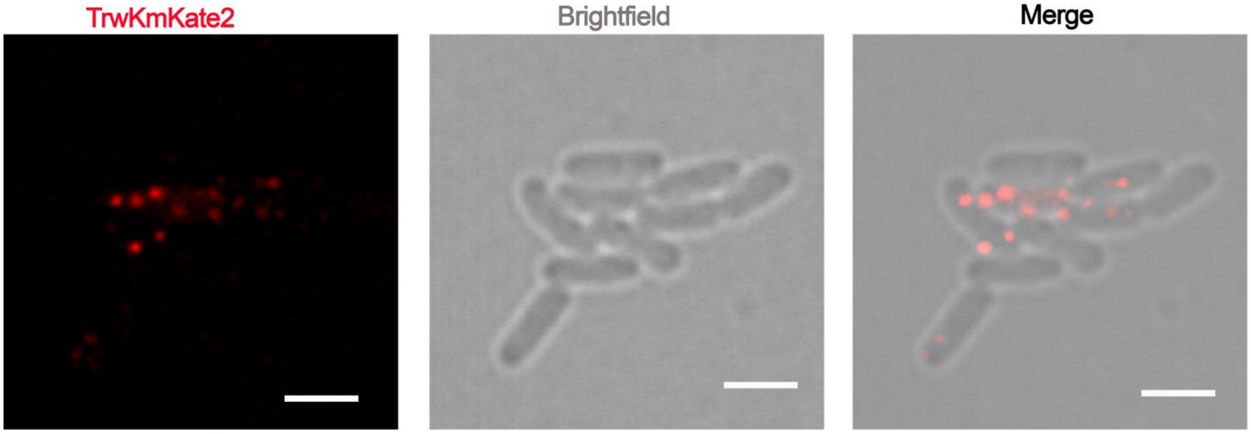

Figure S4. TIRF images of cells expressing TrwKmKate2 fusion protein from its native promotor in plasmid R388. Total Internal Reflection Fluorescence of cells expressing TrwKmKate2 protein were recorded in a Nikon A1 confocal laser microscope equipped with Hamamatsu 9100-C2 camera and a Plan Apochromat TIRF 100x Oil DIC HN2 objective. Under these conditions, the evanescent light produced upon the excitation of a fluorophore located at a distance lower than 200 nm from the cover pad is recorded. This is a standard technique to obtain images of processes that take place in cell membranes.

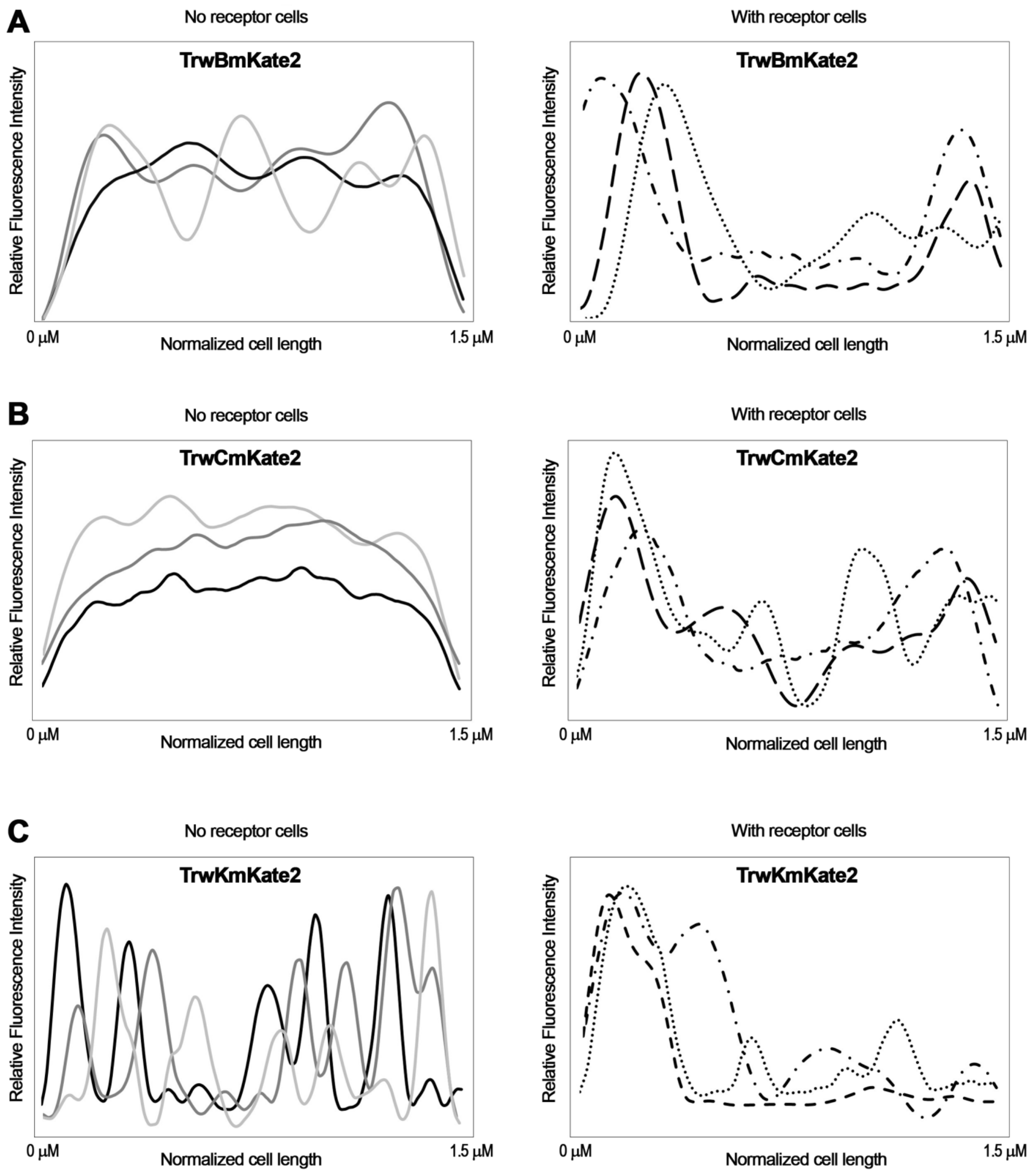

Figure S5. Representative fluorescent intensity profiles in bacterial cells. Images of cells expressing R388_TrwBmKate2 (A), R388_TrwCmKate2 (B) and R388_TrwKmKate2 (C) were analyzed (n=100 for each fluorescent protein). Intensities along the long axis of each cell were normalized to a bacterial size of 1.5 µm. Foci distribution along the cell size was represented in plots in the absence and in the presence of recipient cells (left and right panels, respectively). For clarity, each plot shows only three representative profiles.

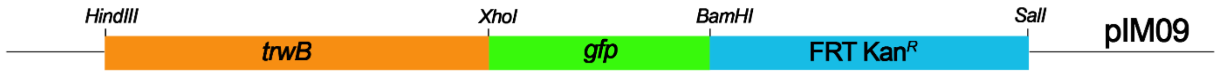

Figure S6. Schematic representation of fluorescent protein constructs. Plasmid pIM09 (I. Matilla, doctoral Thesis, 2011) was used as a template to create all the fluorescent variants fusioned to *trwB*, *trwC* and *trwK* genes. The plasmid contains a fusion of trwB (orange), GFP (green), and kanamicine resistance (blue) genes cloned in a pBR322 derivative vector, using the restriction sites shown in the figure. As indicated in M&M, *trwC* and *trwK* genes were amplified from R388 plasmid by using oligonucleotides flanked by *HindIII* and *XhoI* sites, in order to clone these genes on the same sites in pM09 plasmid. A similar approach was used to clone the different fluorescent variants; in this case into the *XhoI* and *BamH1* restriction sites in pM09 plasmid.
